# Human alterations of the global floodplains 1992–2019

**DOI:** 10.1038/s41597-023-02382-x

**Published:** 2023-07-28

**Authors:** Adnan Rajib, Qianjin Zheng, Charles R. Lane, Heather E. Golden, Jay R. Christensen, Itohaosa I. Isibor, Kris Johnson

**Affiliations:** 1 Hydrology & Hydroinformatics Innovation Lab, Department of Civil Engineering, University of Texas at Arlington, Arlington, Texas USA; 2 U.S. Environmental Protection Agency, Office of Research and Development, Athens, Georgia USA; 3 U.S. Environmental Protection Agency, Office of Research and Development, Cincinnati, Ohio, USA; 4 Department of Environmental Engineering, Texas A&M University, Kingsville, Texas USA; 5 The Nature Conservancy, Minneapolis, Minnesota USA

**Keywords:** Natural hazards, Hydrology, Ecosystem services, Environmental impact

## Abstract

Floodplains provide critical ecosystem services; however, loss of natural floodplain functions caused by human alterations increase flood risks and lead to massive loss of life and property. Despite recent calls for improved floodplain protection and management, a comprehensive, global-scale assessment quantifying human floodplain alterations does not exist. We developed the first publicly available global dataset that quantifies human alterations in 15 million km^2^ floodplains along 520 major river basins during the recent 27 years (1992–2019) at 250-m resolution. To maximize the reuse of our dataset and advance the open science of human floodplain alteration, we developed three web-based programming tools supported with tutorials and step-by-step audiovisual instructions. Our data reveal a significant loss of natural floodplains worldwide with 460,000 km^2^ of new agricultural and 140,000 km^2^ of new developed areas between 1992 and 2019. This dataset offers critical new insights into how floodplains are being destroyed, which will help decision-makers to reinforce strategies to conserve and restore floodplain functions and habitat.

## Background & Summary

Human encroachment of natural floodplains has resulted in altered floodplain land use and levee development, disconnecting and nullifying many floodplain-ecosystem benefits^1–3^. Floodplain functions and their benefits are innumerable. Connectivity between a river and its floodplain^4,5^ is a near-constant, multi-directional feature of river networks that encompasses both surface water expansion and contraction^6^ and groundwater exchange^7,8^. This hydrologic (and hydraulic) coupling – when unaltered – provides opportunities for functions including hydrological and biogeochemical ecosystem services^9,10^. For instance, floodplains provide space for the river to expand during high flows and attenuate flood waters^11,12^. Additionally, when flooding rivers connect via surface water with their floodplains, the increased floodplain roughness (e.g., from riparian vegetation and topography) decreases floodwater velocity and causes sediments and pollutants to settle on the floodplain^13–15^, decreasing pollutant loads in downstream rivers^13,16–20^. Thus, apart from increasing flood risks^21^, floodplain alteration can also decrease drinking and recreational water quality^8,22–25^.

Floodplain alteration places people and property in harm’s way with burdensome financial repercussions^26^. Recent analyses calculated $78 billion in flood-related losses in the conterminous United States for a 100-year flood event in any given year^21^. In fact, Johnson *et al*.^27^ highlight the costs of floodplain alteration, noting that the nearly $8 billion in annual flood losses in the United States alone could be avoided through purchasing and protecting floodplains. However, despite recent calls to improve flood-risk management in the United States^28^ and European countries^29^ and to “…physically separate our activities and infrastructure from floodplains and riparian zones…”^1^, no global assessment quantifying human floodplain alterations exists.

Sustaining floodplain functions and their critical ecosystem benefits demand an accounting of historical trajectories and current trends of human alterations within major floodplains across the globe^30^. Recently, Rajib *et al*.^31^ developed a geospatial dataset of land use change within the Mississippi River Basin floodplains, demonstrating 60 years (1941–2000) of alteration, from relatively natural ecosystems to agricultural and developed land uses. Building off this effort, here we present the first-available global dataset that quantifies human alterations in 15 million km^2^ floodplains along the world’s 520 major river basins. We developed these data using a comprehensive 27-year (1992–2019) analysis of remotely sensed land use change at 250-m resolution.

**Fig. 1:**
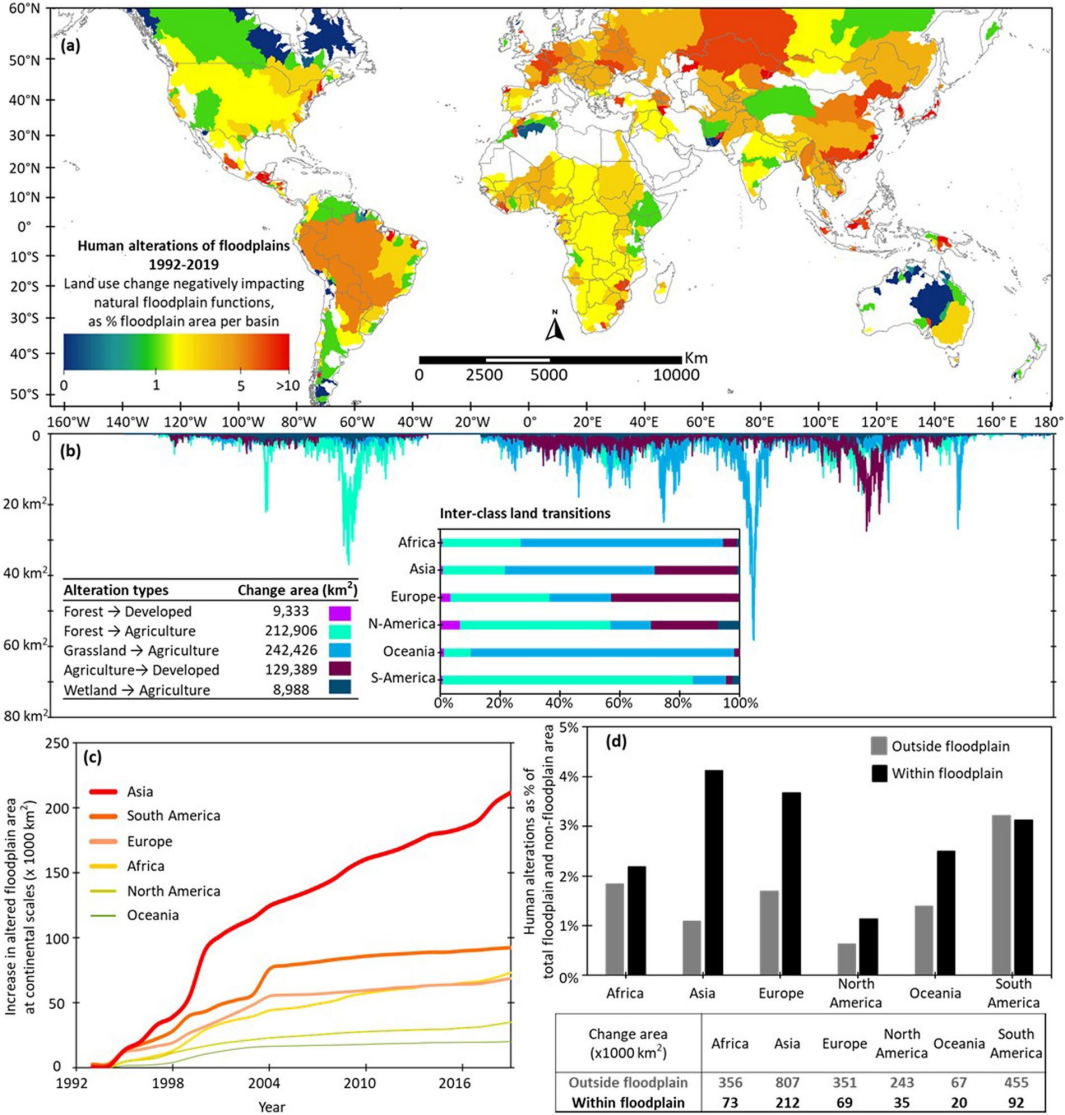
Human alterations of the global floodplains between 1992 and 2019 across 520 major river basins^37^. Human alteration was defined as changes in floodplain land use (e.g., wetland → agriculture) caused by human disturbances that negatively impact floodplain functions. Plot **(a)** maps the degree of floodplain alteration as percent of floodplain area (i.e., total area of “negatively impacting” land use change within the floodplain/total floodplain area of the basin × 100), thus allowing a consistent analysis regardless of the differences in basin sizes and floodplain extents therein. Note, the floodplain dataset used in this analysis (GFPLAIN250m^33^) does not cover deserts and ice-covered regions. Hence, places like northern Africa, Persian Gulf, Tibetan plateau, and the region above 60 degrees north latitude are not shown in plot **(a)**. To identify the characteristic pattern of floodplain alterations, plot **(b)** shows how different alteration types in floodplain land use varied at every 250-m spatial resolution along the latitude, as well as the pattern of inter-class land use transitions at continental scales. Plot **(b)** is further supported by Supplementary Fig. 1 showing how the floodplain alterations at different continents contribute to the overall global floodplain alterations. Plot **(c)** are time-series graphs showing the continuous increase in the area (km^2^) of altered floodplains at continental scales along the 27 years of analysis (1992–2019). Plot **(d)** compares relative degree of alterations within the floodplain and the remaining part of the landscape that is outside the floodplain (i.e., non-floodplain) respectively for every continent. All corresponding data are available for download via HydroShare platform^32^: 10.4211/hs.cdb5fd97e0644a14b22e58d05299f69b.

**Fig. 2:**
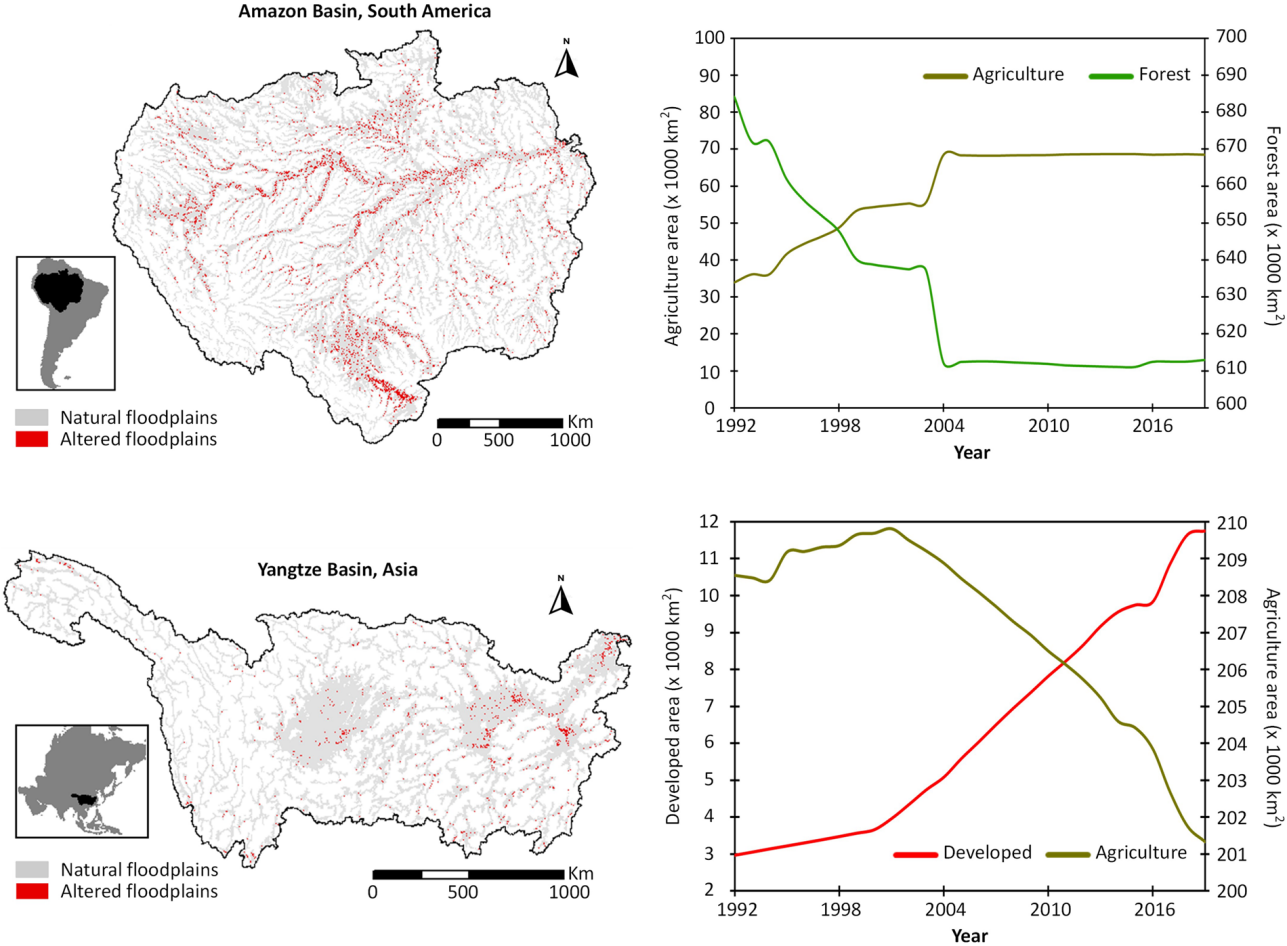
Examples of human alterations of floodplains in two of the world’s major river basins: Amazon in South America and Yangtze in Asia. The corresponding time-series graphs show evidence of underlying human disturbance factors by revealing a nearly *reciprocal* trend of transitions between two dominant land use classes. Other major river basins of the world, e.g., the Great Lakes Basin in North America, Nile River Basin in Africa, Danube River Basin in Europe, Murray River Basin in Oceania show similar examples (Supplementary Figs. 2-3).

This new dataset reveals that the world has lost ~600,000 km^2^ floodplains in 27 years (1992–2019), changing from natural forest, grassland, and wetland conditions to 460,000 km^2^ of new agricultural and 140,000 km^2^ of new developed areas (Fig. 1a,b). The floodplain alteration rate in Asia was particularly high compared to the other continents (Fig. 1c). Further, a *floodplain versus non-floodplain* comparison included in our dataset provides new evidence of greater human disturbance in floodplains relative to non-floodplain portions of the landscape (Fig. 1d), highlighting the need for more focused policy design and implementation. Our dataset additionally reveals specific patterns of land use transitions in some of the major basin floodplains (Fig. 2, Supplementary Figs. 2, 3). For example, within the Amazon River Basin floodplains, increases in agricultural areal extents were nearly proportional to decreases in that of forest (Fig. 2), highlighting Amazon floodplain deforestation. This information provides explicit information on the spatio-temporal dynamics of floodplain alterations in those basins. Our new dataset, along with the corresponding metadata, is available through HydroShare^32^: 10.4211/hs.cdb5fd97e0644a14b22e58d05299f69b. To ensure the maximum reuse of this dataset, we also developed three web-based semi-automatic programming tools partly supported with data-driven tutorials and step-by-step audiovisual instructions.

The scale and rate of land use change in floodplains identified by this dataset is alarming. However, these trends could be halted or reversed, and this dataset could help inform stakeholder-based and government decision-making at various scales. (1) This analysis highlights crucial opportunities for acquisition of vulnerable flood-prone lands recently converted for development or farming. In the United States, for example, the Federal Emergency Management Agency provides funding through several programs for community-based actions, including floodplain buyouts, to reduce future flood risk. This dataset could complement flood loss data and other local information to target buyouts and make effective use of risk mitigation funds. Further, these data could not only inform buyouts of converted lands but could also guide zoning restrictions to limit additional alterations, subsequently decreasing future risk to people and property and mitigating the costs of flood management and recovery. (2) Use of these data could inform policies regarding agricultural land use, to ensure agricultural expansion into flood-prone areas is not incentivized by subsidies or enabled by crop insurance for production in areas likely at high risk for flood loss. (3) With the urgent need to increase climate change resilience and adaptation, in response to extreme floods, local and national governments could use this dataset to target investments in natural or nature-based solutions focused in floodplain restoration.

Overall, the significant alteration of native floodplain forests, wetlands, and grasslands quantified by this analysis suggests increased susceptibility to floods, degraded water quality and habitats, and elevated carbon emissions from biomass and soils throughout the globe^21,24^. Use of this dataset to inform policies that dissuade floodplain alteration and invest in restoration would help reduce risk to people’s health, life, infrastructure, and livelihoods (e.g., farming). These policies would also lead to an essential natural climate solution in the form of intact, functioning floodplain ecosystems and their associated water quality and freshwater and coastal habitats and benefits.

## Methods

### Input data sources

We quantified the human alterations of global floodplains from three input data sources: (1) the GFPLAIN250m global floodplain extent dataset^33^, (2) the European Space Agency’s (ESA) Climate Change Initiative (CCI) annual global land use products^34–36^ from 1992 to 2019, and (3) the Global Runoff Data Centre (GRDC) Major River Basins of the World dataset^37^.

#### Floodplains and non-floodplains definition

Floodplains were defined using GFPLAIN250m dataset. GFPLAIN250m is based on a geomorphic analysis of the Digital Terrain Model (DTM). Its underlying algorithm distinguishes floodplains from surrounding hillslopes as landscape features that have been naturally shaped by accumulated geomorphic effects of past flood events^38–42^. Therefore, this floodplain dataset does not indicate a specific magnitude or return period of flooding (e.g., 100-year floodplains^43,44^). GFPLAIN250m is efficient in mapping floodplains where water-driven erosion and depositional processes are predominant features but have limited efficiency in deserts and ice-covered regions (hence, places like northern Africa, Persian Gulf, Tibetan plateau, and the region above 60 degrees north latitude are not covered by this dataset). The dataset is available in 250-m spatial resolution gridded GeoTIFF format. Areas beyond those identified as floodplains were classified as non-floodplains.

While many sophisticated algorithms, models, and datasets are available for floodplain delineation^43–45^, we selected the GFPLAIN250m dataset considering four factors: spatial resolution, global coverage, well-established literature pool demonstrating the accuracy of the dataset, and importantly a robust algorithm based on the hydrogeomorphological aspects of floodplain formation (not specific to 100-year floods). The GFPLAIN algorithm is openly accessible and has been validated in numerous published research including our previous floodplain alteration study on the Mississippi River Basin^31,46–50^.

#### Land use

The CCI land use dataset is based on the GlobCover unsupervised classification chain^51^ framework. The framework generated global annual land use maps from 1992 to 2019 by using a multi-year and multi-sensor strategy^52^, including observations from Envisat Medium Resolution Imaging Spectrometer (MERIS) (2003–2012), Advanced Very High Resolution Radiometer (AVHRR) (1992–1999), SPOT-Vegetation (SPOT-VGT) (1999–2013), and PROBA-Vegetation (PROBA-V) (2013–2019)^36^. The dataset defines 37 land use classes following the United Nations Land Cover Classification System (UN LCCS)^53,54^. It is available at 300-m spatial resolution in gridded GeoTIFF and NetCDF format. We used the entire range of currently available CCI land use from 1992 to 2019 in our approach to derive the floodplain alteration dataset.

We considered multiple alternative land use datasets^55–58^ while designing our methodology. Using these datasets as the primary inputs in our analysis posed two major challenges. Many datasets encompass intermittent periods of data availability and lack continuity. For example, the cropland extent and change product in the Global Land Analysis & Discovery (GLAD) land use data^56^ is available for 2000–2003 and 2016–2019. While some datasets provide long-term, temporally continuous estimates (e.g., 1982–2016^57^), their limited classification schemes (e.g., vegetation-focused classification used by Song *et al*.^57^ including only tree canopy cover, short vegetation cover, and bare ground cover) exclude important land classes such as wetlands and grasslands which are predominant in global floodplains.

The CCI land use dataset was the best available resource for our targeted scope because (i) it has global coverage with a high spatial resolution (300-m) which is consistent with the spatial resolution of our floodplain extent dataset (250-m), (ii) it uses a detailed land classification scheme, (iii) CCI’s remote sensing-based algorithm as well as the land use product have been validated in numerous published research, including our previous floodplain alteration study on the Mississippi River Basin^31^, and (iv) the dataset is regularly updated to include remotely sensed observations in recent years, thus enabling a pathway to potentially extend our work and develop a continuous floodplain alteration dataset.

#### River basins

The GRDC Major River Basins of the World 2020 was used to define basin boundaries. The dataset was revised and extended from its 2007 edition incorporating data from HydroSHEDS^59,60^ and representing 520 major basins across 977 rivers worldwide^37^. The calculated drainage area per basin ranges from 682 km² up to 6 million km², for the Coatan River Basin and the Amazon River Basin respectively. More than 250 of these 520 basins are transboundary, with more than 90 basins shared by three or more countries. GRDC refers these basins as the “major basins” considering their catchment sizes and hydro-political significance.

We used the Global Human Modification (1990–2017) dataset^61,62^ for verification purposes. This reference dataset is discussed in detail in the Technical Validation section.

### Approach

**Fig. 3:**
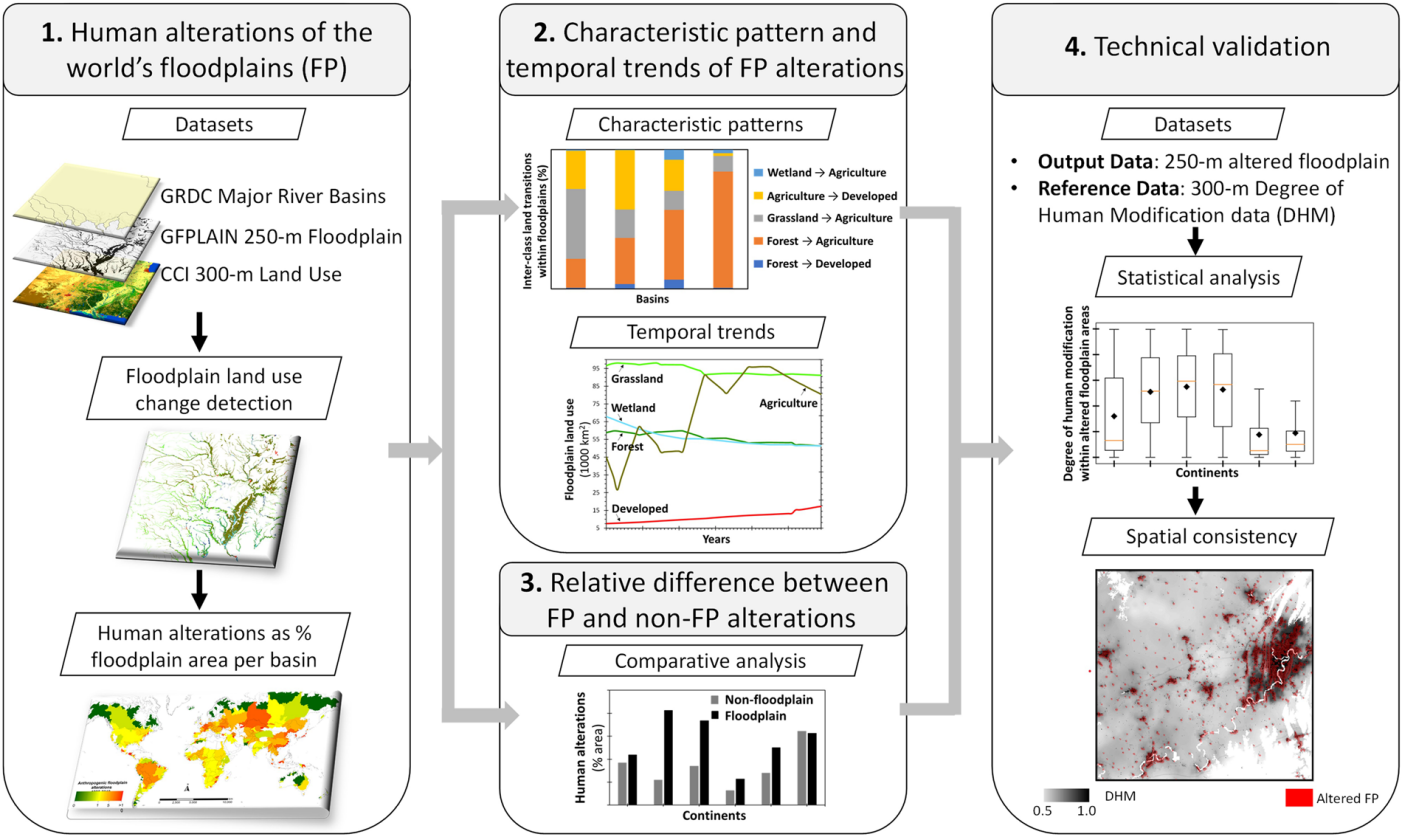
A schematic showing the data development and validation workflow.

In a 3-step approach (Fig. 3), we determined: (1) degree of human alterations in floodplains, (2) characteristic patterns and temporal trends, and (3) relative difference between floodplain and “non-floodplain” landscape alterations. All associated data processing and computation tasks were performed in ArcGIS 10.5 and ENVI 5.1 geospatial platforms.

#### Degree of human alterations in floodplains

We defined “human alterations of floodplains” in terms of changes in floodplain land use that negatively impact floodplain functions. For example, loss of floodplain wetlands and corresponding increase in agricultural or developed areas is a human disturbance, imposes negative impacts (reduced flood water and nutrient storage), and is unlikely to return into a pre-disturbance state (agriculture areas becoming wetlands). We refer to human alterations of floodplains simply as floodplain alterations throughout this paper.

Our definition of human alterations of floodplains is supported by various published research on global change and sustainability science which consider land use change as the direct indicator of human activities and their negative consequences^63–65^. While these functional consequences are complex and often have unknown degree of cause-and-effect relationships among them, use of land use change data is a parsimonious yet highly efficient way to identify the locations and temporal rates at which these consequences might have occurred within the global floodplains over a long period of time (Figs. 1, 2). Although recent studies provided valuable insights into river-floodplain connectivity/integrity by mapping dam and levee locations and/or using statistical tools such as the Connectivity Status Index (CSI)^66–69^, there are three major limitations. Specifically, (i) indicators of connectivity like dams and levees suggest potential altered exchange of water, sediment, nutrients, and habitat along the river network and between the river channel and the adjacent floodplain areas, and therefore do not explicitly indicate floodplain alterations. (ii) The existing river-floodplain connectivity data (e.g., levee locations, CSI values) are unevenly distributed across the globe and static, without providing spatially and temporally continuous estimates essential to understand yearly extent and long-term trends of floodplain alterations. (iii) River-floodplain connectivity does not necessarily involve human drivers (e.g., beaver dams)^70,71^. In short, emerging data of river-floodplain connectivity/integrity are better-suited to study river-floodplain ecosystems in general and may not be applied as the sole indicator of floodplain alterations.

Therefore, our approach to define human floodplain alterations based on land use change data is justified because it offers a quantitative, comprehensive way to measure how floodplains are being altered across space and time^2^, and serves as a direct indicator of the consequences of human disturbance on floodplain ecosystem functions^3,30^. Use of land use change data also offers a scalable pathway to link our floodplain alteration product with numerous existing land planning and policy decision tools^1,72^.

In our previous work on the Mississippi River Basin^31^, we used total area of floodplain land use change as a measure of floodplain alterations. However, because of large variations in floodplain extents across the world’s basins, it is not logical to use the total area of floodplain land use change to compare relative floodplain alterations between two basins. For example, our calculation suggests the same total area of floodplain land use change (945 km^2^) in the Schelde River Basin in Europe and Limpopo River Basin in Africa, although the two basins have very different sizes (19,000 km^2^ and 413,000 km^2^, respectively) and floodplain extents (10,000 km^2^ and 38,000 km^2^, respectively). Clearly, the 945 km^2^ floodplain land use change in these two basins does not express the same magnitude of impact.

To enable a consistent comparison of floodplain alterations by normalizing the differences in basins’ floodplain extents, we calculated the degree of floodplain alteration as percent floodplain extent using this equation:

$$Degree\;of\;floodplain\;alteration=\frac{\sum L{U}_{ChangeAre{a}_{i}}}{\sum F{P}_{TotalExten{t}_{i}}}\times 100$$

Here, $L{U}_{ChangeAre{a}_{i}}$ is the total area of land use change within the floodplain of a basin, $F{P}_{TotalExten{t}_{i}}$ is the total area of floodplain extent within that basin, and *i* indicates floodplain grid-cells. We applied this equation for each of the 520 basins to calculate human alterations of floodplains between 1992 and 2019 (Fig. 1a). The intermediate steps involved in this calculation are elaborated below. Majority of these intermediate steps are adapted from our previous work on the Mississippi River Basin^31^.

##### Land use reclassification

To facilitate easy translation and application of our floodplain alteration dataset across interdisciplinary research, education, and decision-making tools, we reclassified the original 37-class CCI land use dataset into a generic 7-class land use dataset. These seven classes included major land use types: (1) open water, (2) developed area, (3) barren land, (4) forest, (5) grassland, (6) agriculture, and (7) wetland (Supplementary Table 1). Such reclassification greatly reduced the complexity and computational burden in the subsequent steps while preserving the dominant features of the original land use dataset.

##### Extraction of land use within floodplain extents

Because the GFPLAIN250m global floodplain dataset is divided into continents, we used the continent boundary polygons as masks to clip the portion of the reclassified CCI land use within each continent respectively. Next, we resampled the 300-m land use grid-cells to 250-m using the Nearest Neighbor technique to make them consistent with floodplain grid-cells. Finally, we clipped land use with floodplain extent boundary, thus producing a 27-year time-series of annual floodplain land use (1992–2019) at continental scales.

##### Detection of floodplain land use change

Using the 27-year annual floodplain land use time-series, we detected the non-uniqueness of land use grid-cells between two points in time. For each 2-year comparison (i.e., 1992–1993, 1992–1994, and finally 1992–2019), the outcome was a new map with two possible attributes: “1” meaning one unique land use or “no change” and “2” meaning two non-unique land uses or “change” between two points in time.

##### Transition Matrix Analysis

We performed Transition Matrix Analysis^31,73–76^ to quantify how the floodplain land use changed from one class to the other(s) between two years. Among the “change” grid-cells where this analysis suggested human alterations (as defined above; more specifically, forest to developed, forest to agriculture, grassland to agriculture, agriculture to developed, and wetland to agriculture), we calculated $L{U}_{ChangeAre{a}_{i}}$ by multiplying the corresponding total number of grid-cells with the spatial resolution of a single grid-cell (250 × 250 m^2^).

#### Characteristic pattern and temporal trends of floodplain alterations

We aggregated the results of transition analyses to form two-dimensional matrixes at continental scales (Supplementary Tables 2–7). We then used these matrixes to identify the characteristic pattern of floodplain alterations in every continent by calculating the relative proportions of alteration types (Fig. 1b). We also calculated the relative proportions of continents to the global sum of each alteration type, thus identifying how the floodplain alterations at different continents contribute to the overall global floodplain alterations (Supplementary Fig. 1).

Next, we used the 27-year annual floodplain land use time-series (1992–2019) to calculate increases in altered floodplain areas over time in every continent, thus identifying continents that may be needing priority global policy attention for floodplain protection and restoration (Fig. 1c). We also performed a similar temporal analysis at basin scales for six major basins from six different continents, including the Great Lakes Basin in North America, the Amazon River Basin in South America, the Danube River Basin in Europe, the Nile River Basin in Africa, the Yangtze River Basin in Asia, and the Murray River Basin in Australia/Oceania, to provide an explicit understanding of the temporal dynamics of floodplain alterations in those basins (Fig. 2; Supplementary Figs. 2, 3).

#### Relative difference between floodplain and non-floodplain alterations

Our approach to calculate non-floodplain alterations was the same as described in step 1, except we first excluded floodplain extents from the reclassified land use dataset and then applied the subsequent operations on the non-floodplain portion of the basins. We therefore calculated the degree to which floodplain alterations differed from “non-floodplain” landscape alterations between 1992 and 2019 (Fig. 1d).

## Data Records

The global floodplain alteration dataset is available through the HydroShare open geospatial data sharing platform. Our data record also includes all corresponding input data, intermediate calculations, and supporting information. Tables 1, 2 below summarize the file contents. The entire data record can be downloaded as a single zip file from this web link^32^: 10.4211/hs.cdb5fd97e0644a14b22e58d05299f69b.

**Table 1:** Input dataset file descriptions.

Folder Name: Input Data				
ID	Subfolder/File Name	File Type	Content Description	Provenance
1	Global_basin_boundary	GIS shapefile	Boundary polygon of river basins • 520 major river basins across the world	GRDC^37^
2	Global_floodplain	GIS raster	The GFPLAIN250m global floodplain dataset • 250-m grid, GeoTIFF format	Nardi *et al*.^33^
3	Global_floodplain_LU	GIS raster	Remotely sensed land use dataset • Clipped for floodplain extents • Modified to have 7 generic land use classes • One corresponding dataset for each of the 27 years from 1992 to 2019 • The original 300-m data resampled into 250-m, GeoTIFF format	European Space Agency^34–36^
4	Reference_DegreeOfHumanModification	GIS raster	A recently developed global human modification index used as a reference dataset for validation purposes • High modification only (degree of human modification ≥ 0.5) • The original 300-m data resampled into 250-m, GeoTIFF format	Theobald *et al*.^61,62^

**Table 2:** Output dataset file descriptions.

Folder Name: Output Data				
ID	Subfolder/File Name	File Type	Content Description	Output Figure/Table
5	World_ClassTransitionMaps	GIS raster; MS Excel	A global map showing inter-class transitions of land use in the floodplains between 1992 and 2019 across the world’s 520 major river basins • Five types of alterations (e.g., wetland → agriculture) that would negatively impact floodplain functions • 250-m, GeoTIFF format Also includes transition matrix tables between 1992 and 2019 for all seven generic land use classes in floodplains at continental scales, provided in MS Excel format.	Supplementary Tables 2–7
6	Human_FloodplainAlterations	GIS shapefile	A global map showing human alterations of floodplains between 1992 and 2019 • Human alterations were expressed as percent floodplain area within a river basin	Fig. 1a
7	World_ClassTransition_LatLong	MS Excel	Total area (km^2^) for each of the five alteration types (see Table 2 Item 5) between 1992 and 2019 • Calculated along the latitude at every 250-m spacing	Fig. 1b; Supplementary Fig. 1
8	FloodplainAlterations_Continents_Timeseries	MS Excel	Timeseries showing total altered floodplain area (km^2^) at continental scales • 27-year timeseries including data for every year from 1992 to 2019	Fig. 1c
9	AreaChange_In&OutFloodplain	MS Excel	Data showing the relative difference between floodplain and non-floodplain alterations within each continent calculated using input land use dataset (Table 1 Items 2 and 3)	Fig. 1d
10	MajorBasins_Timeseries	MS Excel	Timeseries of total area (km^2^) for each of the 7 generic land use classes across 30 major river basins • 27-year timeseries including data for every year from 1992 to 2019	Fig. 2; Supplementary Figs. 2-3

## Technical Validation

Our input datasets, the GFPLAIN250m floodplain and CCI land use, have been validated in numerous published research^31,33,77–79^, including our previous work on the Mississippi River Basin^31^. We therefore validated our output – the global floodplain alteration dataset – using the global human modification dataset^61,62^ as a reference. This is the only dataset, to our knowledge, that is comparable to ours yet applies independent inputs as well as different underlying concepts and goals, to validate human alterations of global floodplains.

The global human modification verification data are a group of indices indicating where natural terrestrial ecosystems modifications occur based on 14 stressors, e.g., urban development, agricultural expansion, transportation network, power lines, and air pollution, among others. The degree of modification by each stressor combines to a composite index ranging from 0 to 1: “1” is the maximum modification. Stressor assessment is based on remote sensing data (e.g., CCI land use^35,80^, OpenStreetMap^81^, night-time flares^82,83^), traditionally mapped cartographic features (e.g., World Resources Institute Global Power Plant Database^84^, Emission Database for Global Atmospheric Research^85^), and existing stressor classification levels (e.g., the Direct Threats Classification v2^86^). The global human modification dataset is available in 300-m gridded GeoTIFF format for years 1990, 2000, 2010, 2015, and 2017.

**Fig. 4:**
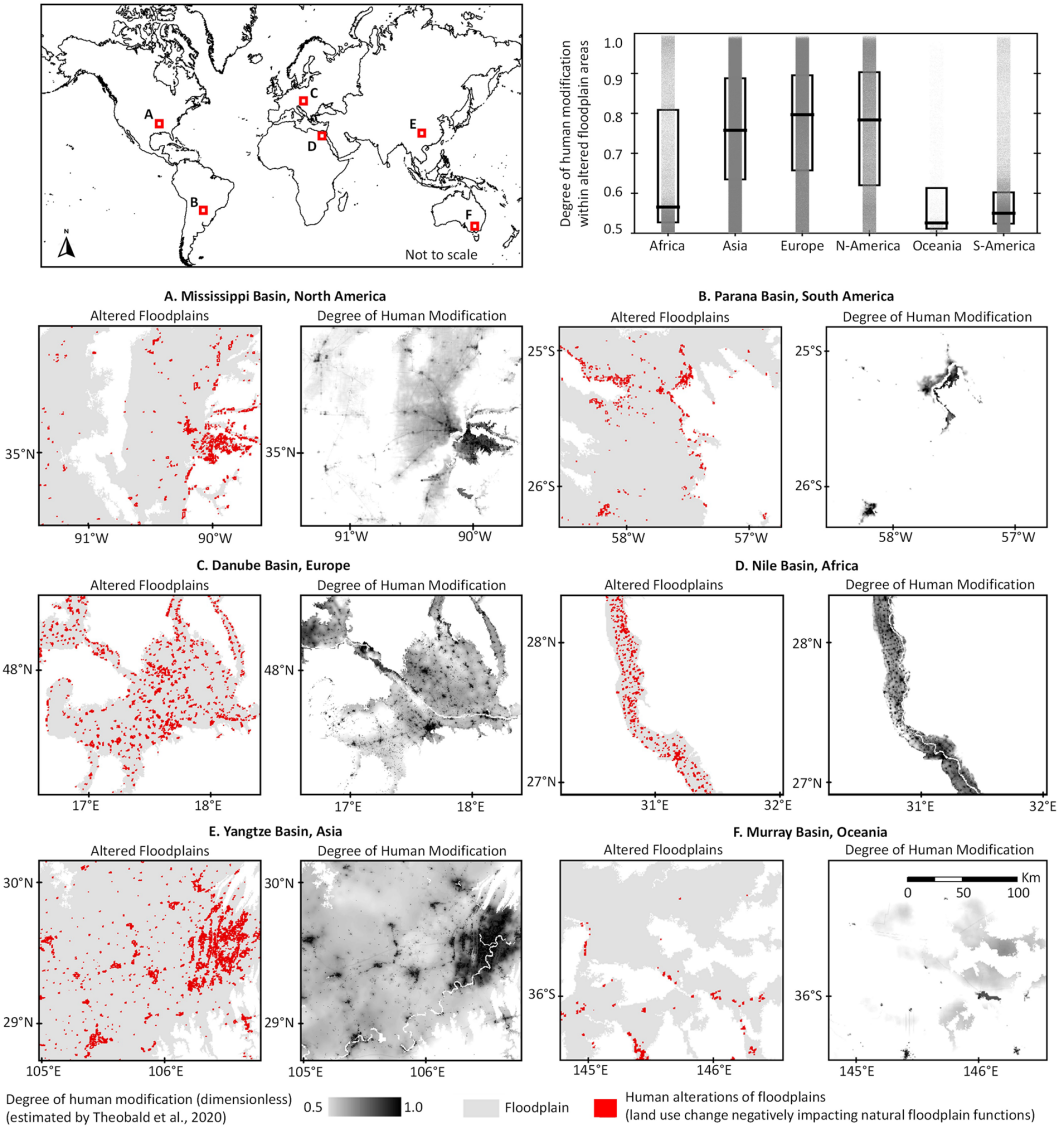
Consistency between the floodplain alteration dataset and a global human modification dataset^61^. The plot shows a parsimonious statistical measure of consistency at continental scales where each dot represents the human modification value for every 250-m altered floodplain grid-cell, and the box represents corresponding 75th percentile, median, and 25th percentile values across continents. The maps are the qualitative measures of spatial resemblance between the two datasets. Because the human modification dataset is not continuous (developed for specific years, e.g., 1990, 2000, 2010, 2015, and 2017), the data presented here are representative of the conditions in year 2017 (i.e., floodplain alterations in 2017 estimated with respect to 1992 are compared with the human modification dataset of 2017^62^).

We used the 2017 human modification dataset to compare with floodplain alterations in 2017 (estimated with respect to 1992, which falls within our 27-year (1992–2019) annual land use change analysis). Because the two datasets are conceptually different, were developed using different methodology, and targeted different goals and outputs, complex statistical tests comparing the two would not be meaningful. Therefore, our verification focused on parsimonious statistical calculations to justify physical consistency and qualitative visual interpretation to understand semantic relatedness^87^ between the two datasets. Specifically, we first extracted human modification values for the altered floodplain grid-cells at continental scales. We then calculated the median, 25^th^ percentile and 75^th^ percentile of human modification data across those grid-cells (Fig. 4). Results suggest that our altered floodplain grid-cells correspond to considerably high human modification values (except Oceania), thus indicating the physical consistency of our data with an independent reference estimate. Next, we randomly selected six floodplain locations (4,000–30,000 km^2^) from six continents and visually inspected their one-on-one resemblance. The high visual resemblance between the two independent datasets across randomly selected locations (Fig. 4) indicates the robust spatial reasoning of our methodology.

## Usage Notes

**Fig. 5:**
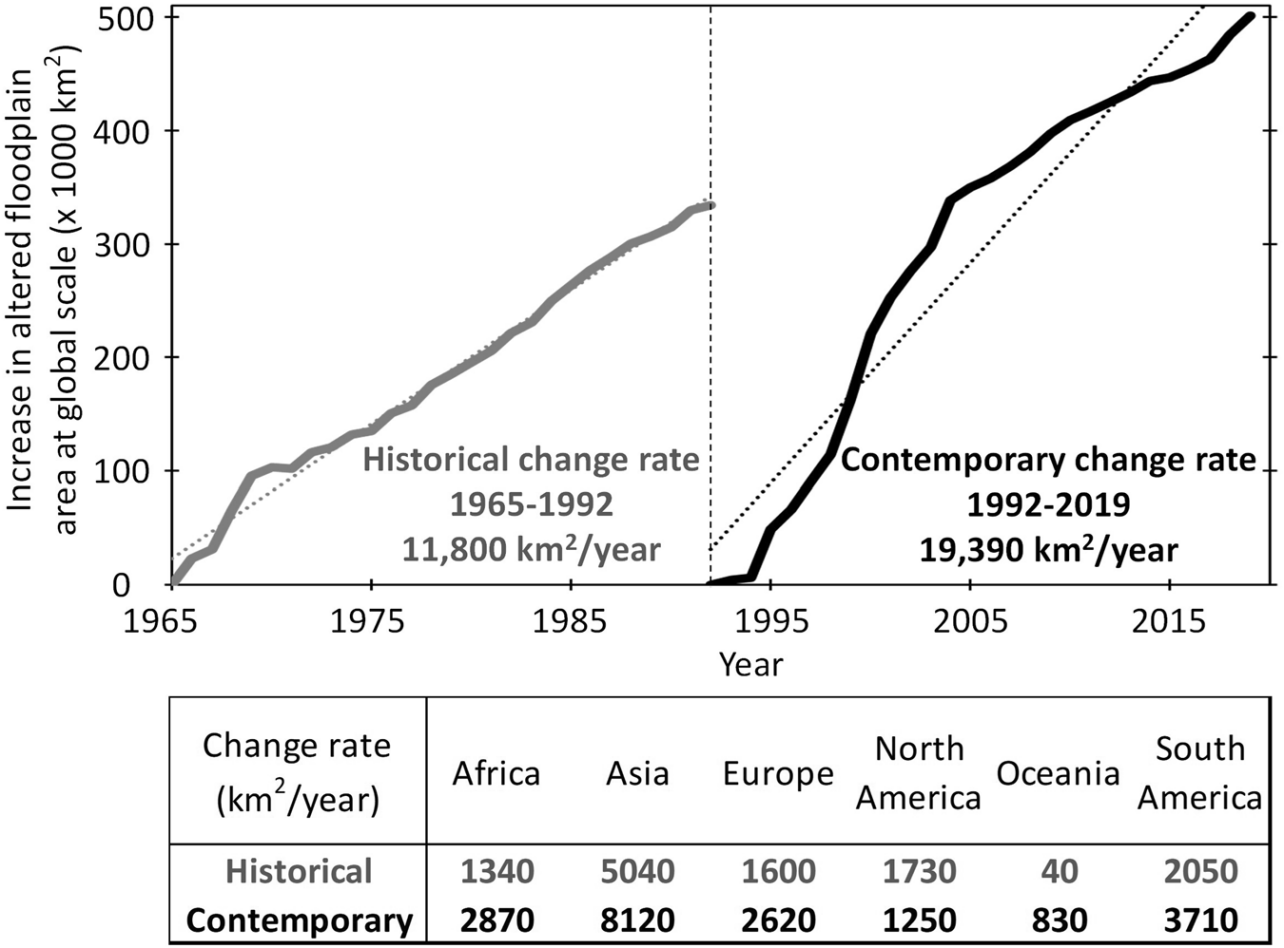
Example demonstrating how the global floodplain alteration dataset can be reused for new applications. The plots graphically compare floodplain alteration rates during the past 27 years (1965–1992; based on a 1-km global land use dataset^88^) and the recent 27 years (1992–2019; based on the 0.25-km output dataset presented here). The change rates (km^2^/year) were calculated with respect to the corresponding base years 1965 and 1992.

The multi-faceted information provided by our Global Floodplain Alteration dataset, e.g., forest to agriculture constituting 83% of floodplain alterations in South America (Fig. 1b) or North America being responsible for 34% of total wetland to agriculture transitions along the global floodplains (Supplementary Fig. 1), are readily useful for making robust policy decisions. However, here we present an additional example demonstrating how some of these information can be reused and integrated into broader research and policy decision workflows. Specifically, we calculated a historical floodplain alteration rate (1965–1992) based on a coarse-resolution (1-km) global land use dataset that hindcasts back to 1960s^88^. We then compared this historical floodplain alteration rate (1965–1992) with a contemporary floodplain alteration rate (1992–2009) based on our dataset at finer resolution (0.25-km). Such a comparison of floodplain alteration rates during the past 27 years (1965–1992) and recent 27 years (1992–2019), at global and continental scales, allowed to examine whether and to what extent floodplain alterations have accelerated (or slowed) in the recent years (Fig. 5). This use case did not reveal any specific pattern, meaning a region with high antecedent floodplain alteration rate before 1992 (the baseline year of our analysis) may not continue to show steeper rates of change in the subsequent years (e.g., North America). This example highlights the importance of long-term, temporally continuous analysis of floodplain alteration in addition to mapping a snapshot of floodplain connectivity/integrity^89^.

To enable instantaneous and effective integration of our floodplain alteration dataset into other scientific workflows such as the one exemplified above, we made our dataset, relevant inputs, and underlying methodology Findable, Accessible, Interoperable, and Reusable (FAIR)^90^. We ensure these FAIR properties by developing three semi-automatic programming tools: (1) Floodplain Mapping Tool, (2) Land Use Change Tool, and (3) Human Alteration Tool. These tools were designed with different levels of complexity to support both the *general users* and *advanced users*.

By gathering user-inputs on geographical extent, each of these tools automatically conducts a set of computation steps in Python programming language and that entirely in Google’s web-based high-performance computing platform called Google Colaboratory. Users will be able to run these tools on an interactive web browser without having to write any new code, deal with GIS software, and manually download and process input datasets, and do computations in personal computers. More importantly, these tools are scalable, meaning advanced users can modify these tools and/or make them interoperate directly with other existing tools, thereby promoting the Open Science principles for cross-disciplinary floodplain research, management, and conservation-restoration decisions.

Links to these tools are provided in the Code Availability section. Users, depending on their objectives, can run these tools in a sequence and answer the following questions.

### How can we identify floodplains?

The Floodplain Mapping Tool will let general users instantaneously access GFPLAIN250m floodplain database and create interactive floodplain maps directly on a web browser. We supplemented this tool by preparing a tutorial via an online data-driven geoscience education platform: https://serc.carleton.edu/hydromodules/steps/246320.html. To further assist the general users, we developed an audiovisual tutorial with step-by-step instructions: https://youtu.be/TgMbkJdALig.

### Where in a floodplain land use changed over time?

The Land Use Change Tool will let general users perform the basic computation steps, including land use reclassification, extraction of land use within floodplain extents, and finally detection of floodplain land use change (see Methods section for detail descriptions). We developed part of this tool during our previous work on the Mississippi River Basin^31^, and subsequently enhanced the tool by making it globally applicable. We supplemented this tool by preparing a tutorial via an online data-driven geoscience education platform: https://serc.carleton.edu/hydromodules/steps/241489.html. The corresponding audiovisual demonstration is available at: https://youtu.be/wH0gif_y15A.

### How to quantify human alterations of floodplains from land use change data?

The Human Alteration Tool is geared mainly towards the advanced users. The tool can perform transition matrix analysis using land use change data (i.e., how one land use class changed into another class between two years), identify the transition types posing negative impacts on floodplain functions (i.e., forest to developed, forest to agriculture, grassland to agriculture, agriculture to developed, and wetland to agriculture), and subsequently calculate degree of human alterations as percent floodplain extent (see Methods section for detail descriptions).

## Supplementary information

Supplementary Information

## Data Availability

The global floodplain alteration dataset was derived entirely through ArcGIS 10.5 and ENVI 5.1 geospatial analysis platforms. To assist in reuse and application of the dataset, we developed additional Python codes aggregated as three web-based tools: Floodplain Mapping Tool: https://colab.research.google.com/drive/1xQlARZXKPexmDInYV-EMoJ-HZxmFL-eW?usp=sharing. Land Use Change Tool: https://colab.research.google.com/drive/1vmIaUCkL66CoTv4rNRIWpJXYXp4TlAKd?usp=sharing. Human Alteration Tool: https://colab.research.google.com/drive/1r2zNJNpd3aWSuDV2Kc792qSEjvDbFtBy?usp=share_link. See Usage Notes section for details.
