# Supplementary material for: Human alterations of the global floodplains 1992–2019
Source: Sci Data. 2023 Jul 28;10:499. doi: 10.1038/s41597-023-02382-x (PMC10382548; doi:10.1038/s41597-023-02382-x)
Supplement: Supplementary file 1 — Supplementary Information [file 41597_2023_2382_MOESM1_ESM.docx]

**Supplementary information**

**for**

**Human alterations of the global floodplains 1992-2019**

Published in the *Scientific Data*

Adnan Rajib^1^, Qianjin Zheng^1^, Charles R. Lane^2^, Heather E. Golden^3^, Jay R. Christensen^3^,

Itohaosa I. Isibor^4^, Kris Johnson^5^

1. Hydrology & Hydroinformatics Innovation Lab, Department of Civil Engineering, University of Texas at Arlington, Texas, USA

2. U.S. Environmental Protection Agency, Office of Research and Development, Athens, Georgia, USA

3. U.S. Environmental Protection Agency, Office of Research and Development, Cincinnati, Ohio, USA

4. Department of Environmental Engineering, Texas A&M University-Kingsville, Texas, USA

5. The Nature Conservancy, Minneapolis, Minnesota, USA

Corresponding author: Adnan Rajib ([adnan.rajib@uta.edu](mailto:adnan.rajib@uta.edu))

**Table of Contents**

[**Supplementary Tables** 3](#_Toc120697602)

[Table 1. 3](#_Toc120697603)

[Table 2. 4](#_Toc120697604)

[Table 3. 5](#_Toc120697605)

[Table 4. 6](#_Toc120697606)

[Table 5. 7](#_Toc120697607)

[Table 6. 8](#_Toc120697608)

[Table 7. 9](#_Toc120697609)

[**Supplementary Figures** 10](#_Toc120697610)

[Figure 1. 10](#_Toc120697611)

[Figure 2. 11](#_Toc120697612)

[Figure 3. 12](#_Toc120697613)

[**References** 13](#_Toc120697614)

# **Supplementary Tables**

**Table 1.** Reclassification scheme of the remotely sensed CCI land use data^1^. The 37 original classes were reclassified to 7 generic classes following Intergovernmental Panel on Climate Change (IPCC) guidelines^2,3^.

| **Original Class** | **New Class** | **Old Value** | **New Value** |
| --- | --- | --- | --- |
| Cropland | Agriculture | 10 | 6 |
| Herbaceous cover | Agriculture | 11 | 6 |
| Tree or shrub cover | Agriculture | 12 | 6 |
| Cropland irrigated or post-flooding | Agriculture | 20 | 6 |
| Mosaic cropland (>50%) /natural vegetation (tree shrub herbaceous cover) (<50%) | Agriculture | 30 | 6 |
| Mosaic natural vegetation (tree shrub herbaceous cover) (>50%) /cropland (<50%) | Agriculture | 40 | 6 |
| Tree cover broadleaved evergreen closed to open (>15%) | Forest | 50 | 4 |
| Tree cover broadleaved deciduous closed to open (>15%) | Forest | 60 | 4 |
| Tree cover broadleaved deciduous closed (>40%) | Forest | 61 | 4 |
| Tree cover broadleaved deciduous open (15-40%) | Forest | 62 | 4 |
| Tree cover needleleaved evergreen closed to open (>15%) | Forest | 70 | 4 |
| Tree cover needleleaved evergreen closed (>40%) | Forest | 71 | 4 |
| Tree cover needleleaved evergreen open (15-40%) | Forest | 72 | 4 |
| Tree cover needleleaved deciduous closed to open (>15%) | Forest | 80 | 4 |
| Tree cover needleleaved deciduous closed (>40%) | Forest | 81 | 4 |
| Tree cover needleleaved deciduous open (15-40%) | Forest | 82 | 4 |
| Tree cover mixed leaf type (broadleaved and needleleaved) | Forest | 90 | 4 |
| Mosaic tree and shrub (>50%) / herbaceous cover (<50%) | Forest | 100 | 4 |
| Mosaic herbaceous cover (>50%) / tree and shrub (<50%) | Grassland | 110 | 5 |
| Shrubland | Grassland | 120 | 5 |
| Shrubland evergreen | Grassland | 121 | 5 |
| Shrubland deciduous | Grassland | 122 | 5 |
| Grassland | Grassland | 130 | 5 |
| Lichens and mosses | Grassland | 140 | 5 |
| Sparse vegetation (tree shrub herbaceous cover) (<15%) | Grassland | 150 | 5 |
| Sparse tree (<15%) | Grassland | 151 | 5 |
| Sparse shrub (<15%) | Grassland | 152 | 5 |
| Sparse herbaceous cover (<15%) | Grassland | 153 | 5 |
| Tree cover flooded fresh or brackish water | Forest | 160 | 4 |
| Tree cover flooded saline water | Forest | 170 | 4 |
| Shrub or herbaceous cover flooded fresh/saline/brackish water | Wetland | 180 | 7 |
| Urban areas | Developed | 190 | 2 |
| Bare areas | Barren | 200 | 3 |
| Consolidated bare areas | Barren | 201 | 3 |
| Unconsolidated bare areas | Barren | 202 | 3 |
| Water bodies | Water | 210 | 1 |
| Permanent snow and ice | Water | 220 | 1 |

**Table 2.** Inter-class transition (km^2^) matrix across seven generic classes of floodplain land use in Asia between 1992 and 2019. The diagonal entries indicate the total size of persistent land use types, whereas other entries indicate the transition size of one land use type into another. See Table 1 by Rajib et al.^4^ for detailed explanation of the transition matrix.

|  | | **Land use in floodplain in year 1992 across Asia** **(km^2^)** | | | | | | | **Total area in 2019** |
| --- | --- | --- | --- | --- | --- | --- | --- | --- | --- |
|  |  | **Water** | **Developed** | **Barren** | **Forest** | **Grassland** | **Agriculture** | **Wetland** |  |
| **Land use in floodplain in year 2019 across Asia** **(km^2^)** | **Water** | 482,552 | 0 | 3,697 | 11,969 | 8,773 | 10,585 | 2,299 | 519,875 |
|  | **Developed** | 935 | 39,883 | 1,732 | 2,384 | 13,761 | 78,183 | 731 | 137,609 |
|  | **Barren** | 4,600 | 0 | 238,147 | 509 | 19,209 | 1,950 | 11 | 264,427 |
|  | **Forest** | 1,706 | 0 | 156 | 964,407 | 18,748 | 32,697 | 23,991 | 1,041,705 |
|  | **Grassland** | 5,719 | 0 | 16,841 | 22,329 | 1,010,023 | 12,667 | 1,467 | 1,069,045 |
|  | **Agriculture** | 5,308 | 0 | 6,931 | 58,883 | 142,139 | 2,924,670 | 2,267 | 3,140,199 |
|  | **Wetland** | 5,554 | 0 | 8 | 18,232 | 830 | 3,419 | 264,954 | 292,997 |
| **Total area in 1992** | | 506,373 | 39,883 | 267,511 | 1,078,714 | 1,213,483 | 3,064,171 | 295,720 |  |
| **Change in each land class between 1992 and 2019** | | 23,821 | 0 | 29,364 | 114,307 | 203,460 | 139,501 | 30,766 |  |
| **Difference** | | 13,502 | 97,726 | -3,085 | -37,009 | -144,438 | 76,028 | -2,723 |  |

**Table 3.** Inter-class transition (km^2^) matrix across seven generic classes of floodplain land use in Africa between 1992 and 2019. The diagonal entries indicate the total size of persistent land use types, whereas other entries indicate the transition size of one land use type into another. See Table 1 by Rajib et al.^4^ for detailed explanation of the transition matrix.

|  | | **Land use in floodplain in year 1992 across Africa (km^2^)** | | | | | | | **Total area in 2019** |
| --- | --- | --- | --- | --- | --- | --- | --- | --- | --- |
|  |  | **Water** | **Developed** | **Barren** | **Forest** | **Grassland** | **Agriculture** | **Wetland** |  |
| **Land use in floodplain in year 2019 across Africa (km^2^)** | **Water** | 119,019 | 0 | 1,699 | 1,933 | 1,430 | 1,860 | 2,843 | 128,783 |
|  | **Developed** | 94 | 3,933 | 465 | 456 | 1,775 | 3,614 | 157 | 10,495 |
|  | **Barren** | 791 | 0 | 655,557 | 165 | 8,795 | 296 | 34 | 665,638 |
|  | **Forest** | 742 | 0 | 288 | 525,039 | 35,559 | 17,878 | 4,878 | 584,383 |
|  | **Grassland** | 1,099 | 0 | 45,364 | 13,507 | 1,207,620 | 7,578 | 285 | 1,275,452 |
|  | **Agriculture** | 1,251 | 0 | 1,379 | 21,084 | 54,056 | 824,707 | 888 | 903,364 |
|  | **Wetland** | 3,420 | 0 | 26 | 9,915 | 1,105 | 1,755 | 272,793 | 289,013 |
| **Total area in 1992** | | 126,417 | 3,933 | 704,777 | 572,098 | 1,310,340 | 857,687 | 281,877 |  |
| **Change in each land class between 1992 and 2019** | | 7,398 | 0 | 49,220 | 47,059 | 102,720 | 32,980 | 9,084 |  |
| **Difference** | | 2,366 | 6,562 | -39,139 | 12,285 | -34,888 | 45,677 | 7,136 |  |

**Table 4.** Inter-class transition (km^2^) matrix across seven generic classes of floodplain land use in Europe between 1992 and 2019. The diagonal entries indicate the total size of persistent land use types, whereas other entries indicate the transition size of one land use type into another. See Table 1 by Rajib et al. ^4^ for detailed explanation of the transition matrix.

|  | | **Land use in floodplain in year 1992 across Europe (km^2^)** | | | | | | | **Total area in 2019** |
| --- | --- | --- | --- | --- | --- | --- | --- | --- | --- |
|  |  | **Water** | **Developed** | **Barren** | **Forest** | **Grassland** | **Agriculture** | **Wetland** |  |
| **Land use in floodplain in year 2019 across Europe (km^2^)** | **Water** | 183,702 | 0 | 306 | 5,342 | 546 | 3,819 | 283 | 193,999 |
|  | **Developed** | 391 | 48,509 | 1,001 | 2,886 | 2,853 | 35,929 | 317 | 91,886 |
|  | **Barren** | 238 | 0 | 2,389 | 51 | 56 | 25 | 0 | 2,759 |
|  | **Forest** | 999 | 0 | 24 | 418,676 | 2,504 | 37,043 | 1,366 | 460,611 |
|  | **Grassland** | 427 | 0 | 206 | 5,282 | 245,767 | 5,518 | 95 | 257,295 |
|  | **Agriculture** | 1,034 | 0 | 59 | 27,896 | 17,249 | 1,094,231 | 256 | 1,140,724 |
|  | **Wetland** | 681 | 0 | 3 | 8,067 | 257 | 1,294 | 51,113 | 61,415 |
| **Total area in 1992** | | 187,472 | 48,509 | 3,988 | 468,200 | 269,233 | 1,177,858 | 53,429 |  |
| **Change in each land class between 1992 and 2019** | | 3,770 | 0 | 1,599 | 49,523 | 23,465 | 83,627 | 2,316 |  |
| **Difference** | | 6,526 | 43,377 | -1,230 | -7,588 | -11,937 | -37,134 | 7,986 |  |

**Table 5.** Inter-class transition (km^2^) matrix across seven generic classes of floodplain land use in North America between 1992 and 2019. The diagonal entries indicate the total size of persistent land use types, whereas other entries indicate the transition size of one land use type into another. See Table 1 by Rajib et al.^4^ for detailed explanation of the transition matrix.

|  | | **Land use in floodplain in year 1992 across North America (km^2^)** | | | | | | | **Total area in 2019** |
| --- | --- | --- | --- | --- | --- | --- | --- | --- | --- |
|  |  | **Water** | **Developed** | **Barren** | **Forest** | **Grassland** | **Agriculture** | **Wetland** |  |
| **Land use in floodplain in year 2019 across North America (km^2^)** | **Water** | 879,015 | 0 | 583 | 24,581 | 2,596 | 2,166 | 1,781 | 910,722 |
|  | **Developed** | 272 | 29,055 | 384 | 2,761 | 4,370 | 9,379 | 659 | 46,879 |
|  | **Barren** | 916 | 0 | 8,216 | 1,303 | 60 | 10 | 57 | 10,563 |
|  | **Forest** | 7,711 | 0 | 107 | 1,170,115 | 21,433 | 14,107 | 16,220 | 1,229,693 |
|  | **Grassland** | 736 | 0 | 38 | 21,679 | 345,047 | 2,260 | 2,782 | 372,543 |
|  | **Agriculture** | 304 | 0 | 10 | 21,087 | 5,709 | 725,726 | 3,046 | 755,883 |
|  | **Wetland** | 548 | 0 | 12 | 4,447 | 1,852 | 3,379 | 222,162 | 232,401 |
| **Total area in 1992** | | 889,502 | 29,055 | 9,350 | 1,245,973 | 381,068 | 757,027 | 246,708 |  |
| **Change in each land class between 1992 and 2019** | | 10,487 | 0 | 1,134 | 75,859 | 36,021 | 31,301 | 24,546 |  |
| **Difference** | | 21,220 | 17,824 | 1,213 | -16,280 | -8,525 | -1,144 | -14,307 |  |

**Table 6.** Inter-class transition (km^2^) matrix across seven generic classes of floodplain land use in South America between 1992 and 2019. The diagonal entries indicate the total size of persistent land use types, whereas other entries indicate the transition size of one land use type into another. See Table 1 by Rajib et al.^4^ for detailed explanation of the transition matrix.

|  | | **Land use in floodplain in year 1992 across South America (km^2^)** | | | | | | | **Total area in 2019** |
| --- | --- | --- | --- | --- | --- | --- | --- | --- | --- |
|  |  | **Water** | **Developed** | **Barren** | **Forest** | **Grassland** | **Agriculture** | **Wetland** |  |
| **Land use in floodplain in year 2019 across South America (km^2^)** | **Water** | 193,835 | 0 | 166 | 31,892 | 3,787 | 2,271 | 9,230 | 241,179 |
|  | **Developed** | 45 | 5,387 | 140 | 673 | 1,143 | 2,032 | 195 | 9,614 |
|  | **Barren** | 557 | 0 | 127,486 | 662 | 2,922 | 104 | 24 | 131,755 |
|  | **Forest** | 1,509 | 0 | 24 | 1,106,017 | 24,545 | 20,070 | 37,980 | 1,190,144 |
|  | **Grassland** | 3,829 | 0 | 5,120 | 59,454 | 675,058 | 2,941 | 1,915 | 748,318 |
|  | **Agriculture** | 881 | 0 | 304 | 82,707 | 11,035 | 513,909 | 2,519 | 611,355 |
|  | **Wetland** | 4,987 | 0 | 6 | 60,562 | 3,505 | 1,947 | 366,347 | 437,353 |
| **Total area in 1992** | | 205,643 | 5,387 | 133,246 | 1,341,967 | 721,994 | 543,273 | 418,210 |  |
| **Change in each land class between 1992 and 2019** | | 11,808 | 0 | 5,760 | 235,950 | 46,936 | 29,364 | 51,863 |  |
| **Difference** | | 35,536 | 4,228 | -1,491 | -151,822 | 26,324 | 68,082 | 19,143 |  |

**Table 7.** Inter-class transition (km^2^) matrix across seven generic classes of floodplain land use in Oceania between 1992 and 2019. The diagonal entries indicate the total size of persistent land use types, whereas other entries indicate the transition size of one land use type into another. See Table 1 by Rajib et al.^4^ for detailed explanation of the transition matrix.

|  | | **Land use in floodplain in year 1992 across Oceania (km^2^)** | | | | | | | **Total area in 2019** |
| --- | --- | --- | --- | --- | --- | --- | --- | --- | --- |
|  |  | **Water** | **Developed** | **Barren** | **Forest** | **Grassland** | **Agriculture** | **Wetland** |  |
| **Land use in floodplain in year 2019 across Oceania (km^2^)** | **Water** | 8,064 | 0 | 38 | 281 | 634 | 247 | 86 | 9,351 |
|  | **Developed** | 11 | 1,695 | 11 | 173 | 442 | 243 | 10 | 2,584 |
|  | **Barren** | 2 | 0 | 13,846 | 20 | 1,565 | 34 | 13 | 15,479 |
|  | **Forest** | 84 | 0 | 3 | 83,537 | 10,956 | 1,474 | 206 | 96,259 |
|  | **Grassland** | 398 | 0 | 1,000 | 6,967 | 634,648 | 1,033 | 85 | 644,130 |
|  | **Agriculture** | 184 | 0 | 1 | 1,243 | 12,236 | 173,367 | 11 | 187,042 |
|  | **Wetland** | 37 | 0 | 3 | 628 | 244 | 73 | 40,971 | 41,956 |
| **Total area in 1992** | | 8,780 | 1,695 | 14,902 | 92,849 | 660,725 | 176,470 | 41,381 |  |
| **Change in each land class between 1992 and 2019** | | 715 | 0 | 1,056 | 9,312 | 26,078 | 3,103 | 410 |  |
| **Difference** | | 571 | 889 | 578 | 3,411 | -16,595 | 10,572 | 575 |  |

# **Supplementary Figures**

**Figure 1.** Graphic how the floodplain alterations at different continents contribute to the overall global floodplain alterations (1992-2019), expressed as percentage of every continent for specific alteration types.

**Figure 2.** Examples of human alterations of floodplains in two of the world’s major river basins: Great Lakes Basin in North America and Nile River Basin in Africa. The corresponding time-series graphs show evidence of underlying human disturbance factors by revealing a nearly *reciprocal* trend of transitions between two dominant land use classes. See Supplementary Fig. 3 for similar examples in other major basins of the world.

**Figure 3.** Examples of human alterations of floodplains in two of the world’s major river basins: Danube River Basin in Europe and Murray River Basin in Oceania. The corresponding time-series graphs show evidence of underlying human disturbance factors by revealing a nearly *reciprocal* trend of transitions between two dominant land use classes. See Supplementary Fig. 2 for similar examples in other major basins of the world.

# **References**

1. Climate Change Initiative (CCI) of the European Space Agency, 2018. *CCI-LC products.* <http://maps.elie.ucl.ac.be/CCI/viewer/download.php>
2. European Space Agency. 2017. *Land Cover CCI Product User Guide Version 2. Tech. Rep.* <http://maps.elie.ucl.ac.be/CCI/viewer/download/ESACCI-LC-Ph2-PUGv2_2.0.pdf>.
3. *Land Cover CCI Product User Guide Version 2.1*. Tech. Rep. <https://datastore.copernicus-climate.eu/documents/satellite-land-cover/D5.3.1_PUGS_ICDR_LC_v2.1.x_PRODUCTS_v1.1.pdf> (European Space Agency, 2021).
4. Rajib, A. *et al.* The changing face of floodplains in the Mississippi River Basin detected by a 60-year land use change dataset. *Scientific Data* **8**, 271, <https://doi.org/10.1038/s41597-021-01048-w> (2021).
